# Supplementary material for: Microeukaryotic Communities on the Fruit of Gardenia thunbergia Thunb. with a Focus on Pathogenic Fungi
Source: Pathogens. 2021 May 4;10(5):555. doi: 10.3390/pathogens10050555 (PMC8147784; doi:10.3390/pathogens10050555)
Supplement: Supplementary file 1 [file pathogens-10-00555-s001.zip › pathogens-1202147-supplementary.pdf]

Table S1: Samples used in this study

sample number	Source	sampled material	location	coordinates S	coordinates E
1	*Caesalpinia ferrea* C.Mart. ex Tul.	tree bark	Durban, Sherwood, Straberry Fields	29°49´49.8´´	30°58´04.7´´
2	*Encephalartos ferox* G. Bertol.	leaf scale	Durban, Reservoir hills	29°4´16.6´´	30°56´16.5´´
3	Cement wall	surface	Durban, Chatsworth, Unit 2	29°55´10.3´´	30°55´00.2´´
4	*Syagrus romanzoffiana* (Cham.) Glassman	stem surface bark	Durban, Sherwood, Straberry Fields	29°49´46.5´´	30°58´13.8´´
5	Storm water open cement wall	surface	Durban, Sherwood, Straberry Fields	29°49´48.4´´	30°58´04.1´´
6	Floor cement outside	surface	Durban, Sherwood, Straberry Fields	29°49´47.7´´	30°58´09.5´´
7	*Trichilia emetica* Vahl	bark surface	Durban, University of KwaZulu-Natal (Westville Campus)	29°49´00.6´´	30°56´26.0´´
8	*Codiaeum variegatum* (L.) Rumph. ex A. Juss	bark surface	Durban, Chatsworth, Unit 2	29°55´10.3´´	30°55´00.2´´
9	*Yucca gigantea* Lim.	stem surface	Durban, Sherwood, Straberry Fields	29°49´48.5``	30°58´11.1´´
10 to 16	*Gardenia thunbergia* Thunb.	indehiscent fruits	Durban, Sherwood, Straberry Fields	29°49´50.3´´	30°58´06.9´´

Table S2: Similarity values between the replicates of one sample. Note that, besides from samples Afr_12 and Afr_14, the samples from the *G. thunbergia* Thunb. fruits are very similar in the replicates.

sample	comparison	r value
Afr_1	a ~ b	0.862
Afr_1	a ~ c	0.805
Afr_1	b ~ c	0.94
Afr_2	a ~ b	0.974
Afr_2	a ~ c	0.964
Afr_2	b ~ c	0.93
Afr_3	a ~ b	/
Afr_3	a ~ c	/
Afr_3	b ~ c	/
Afr_4	a ~ b	0.878
Afr_4	a ~ c	0.953
Afr_4	b ~ c	0.859
Afr_5	a ~ b	0.734
Afr_5	a ~ c	0.965
Afr_5	b ~ c	0.745
Afr_6	a ~ b	0.991
Afr_6	a ~ c	0.924
Afr_6	b ~ c	0.878
Afr_7	a ~ b	0.771
Afr_7	a ~ c	0.764
Afr_7	b ~ c	0.998
Afr_8	a ~ b	0.541
Afr_8	a ~ c	0.754
Afr_8	b ~ c	0.878
Afr_9	a ~ b	0.993
Afr_9	a ~ c	0.993
Afr_9	b ~ c	0.999
Afr_10	a ~ b	0.946
Afr_10	a ~ c	0.996
Afr_10	b ~ c	0.924
Afr_11	a ~ b	0.994
Afr_11	a ~ c	0.973
Afr_11	b ~ c	0.979
Afr_12	a ~ b	0.721
Afr_12	a ~ c	0.995
Afr_12	b ~ c	0.729
Afr_13	a ~ b	0.963
Afr_13	a ~ c	0.999
Afr_13	b ~ c	0.967
Afr_14	a ~ b	0.729
Afr_14	a ~ c	0.804
Afr_14	b ~ c	0.954
Afr_15	a ~ b	0.991
Afr_15	a ~ c	/
Afr_15	b ~ c	/
Afr_16	a ~ b	0.954
Afr_16	a ~ c	0.957
Afr_16	b ~ c	0.995
mean		0.899

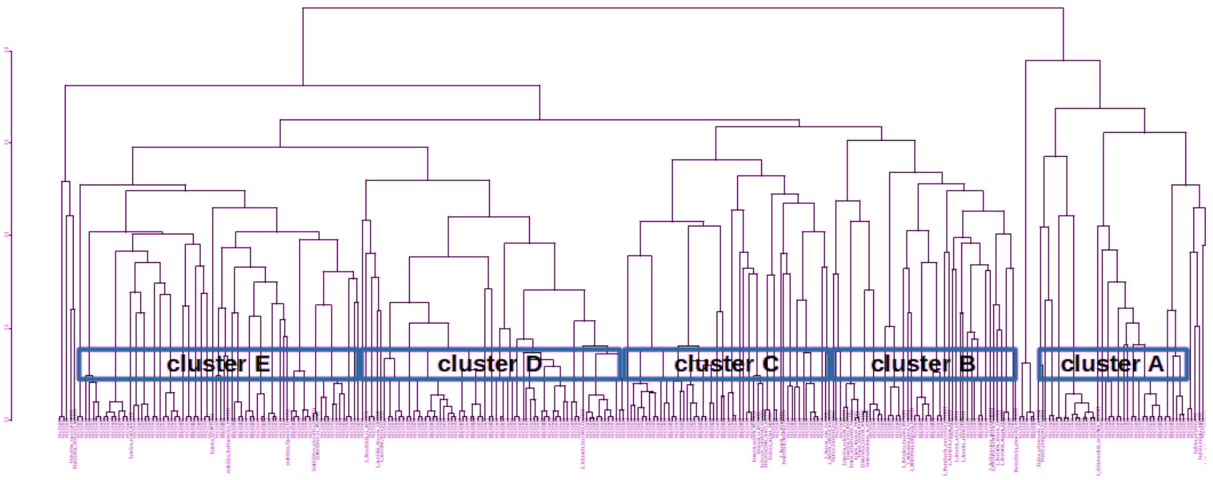

Figure S1: Overview on the clusters based on the dendrogram including all sequences. The remaining sequences not included in clusters A to E are treated as cluster F in the main paper.

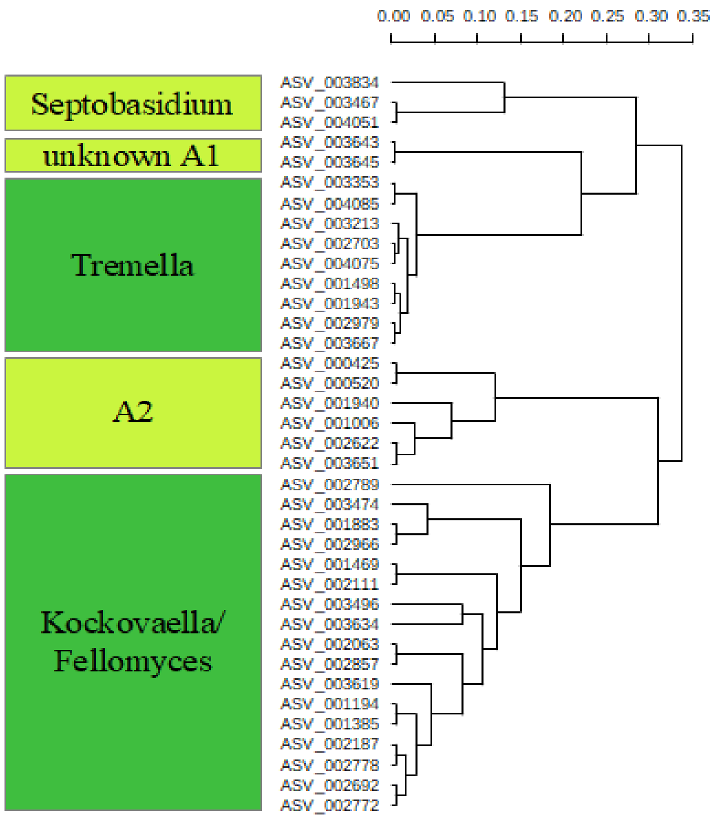

Fig. S2: Dendrogram of the Basidiomycota clade A. Dark green boxes indicate clusters that are very unlikely to contain plant pathogens, light green clusters may contain plant pathogens. The level of difference is given in the scale.

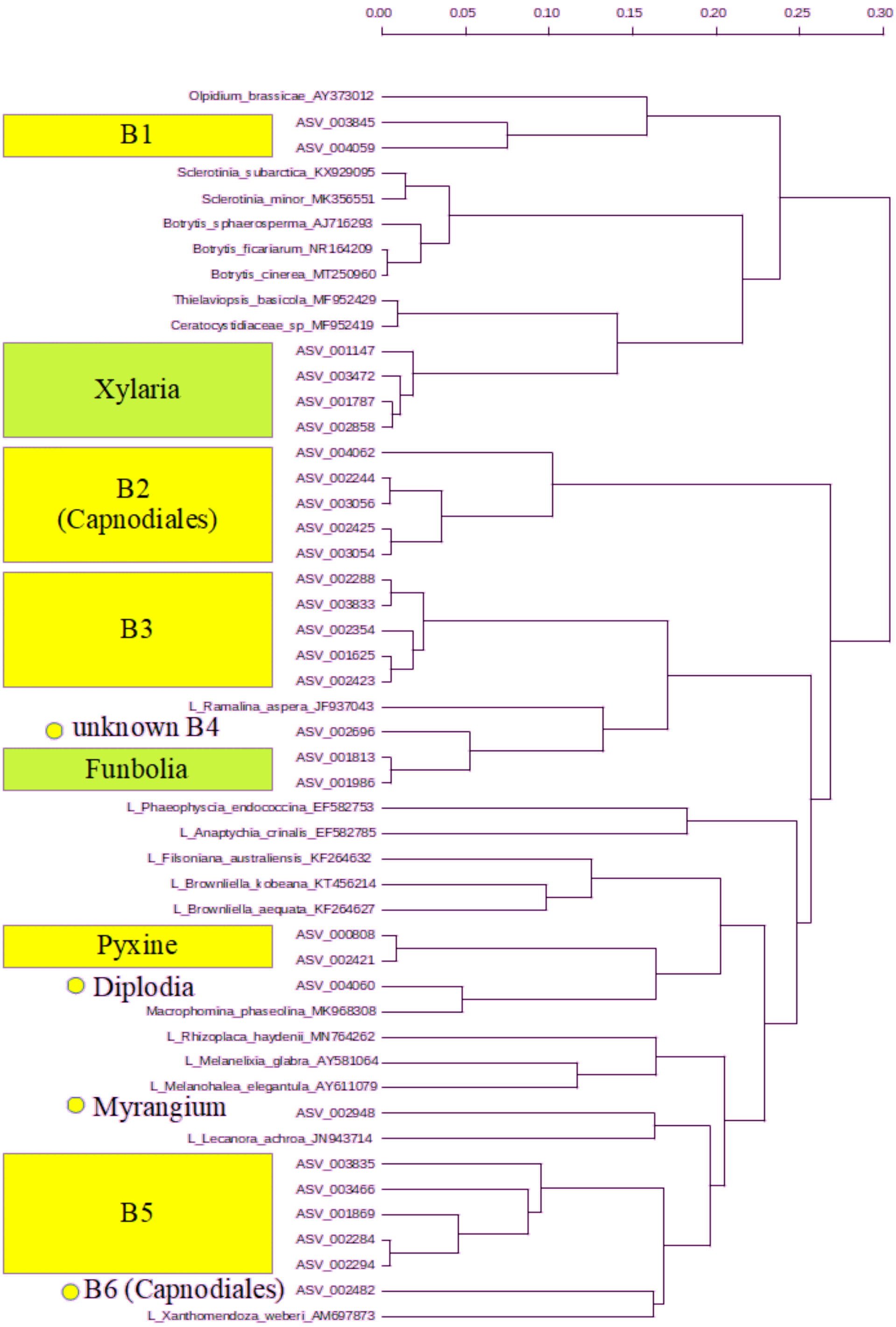

Fig. S3: Dendrogram of cluster B. Light green clusters that are unlikely to contain plant pathogens, yellow clusters can not be classified regarding their pathogenic potential. Singleton sequences distinct from other clusters if those are not known or determined as another taxon of the next neighbouring clade are marked with a dot. The level of difference is given in the scale.

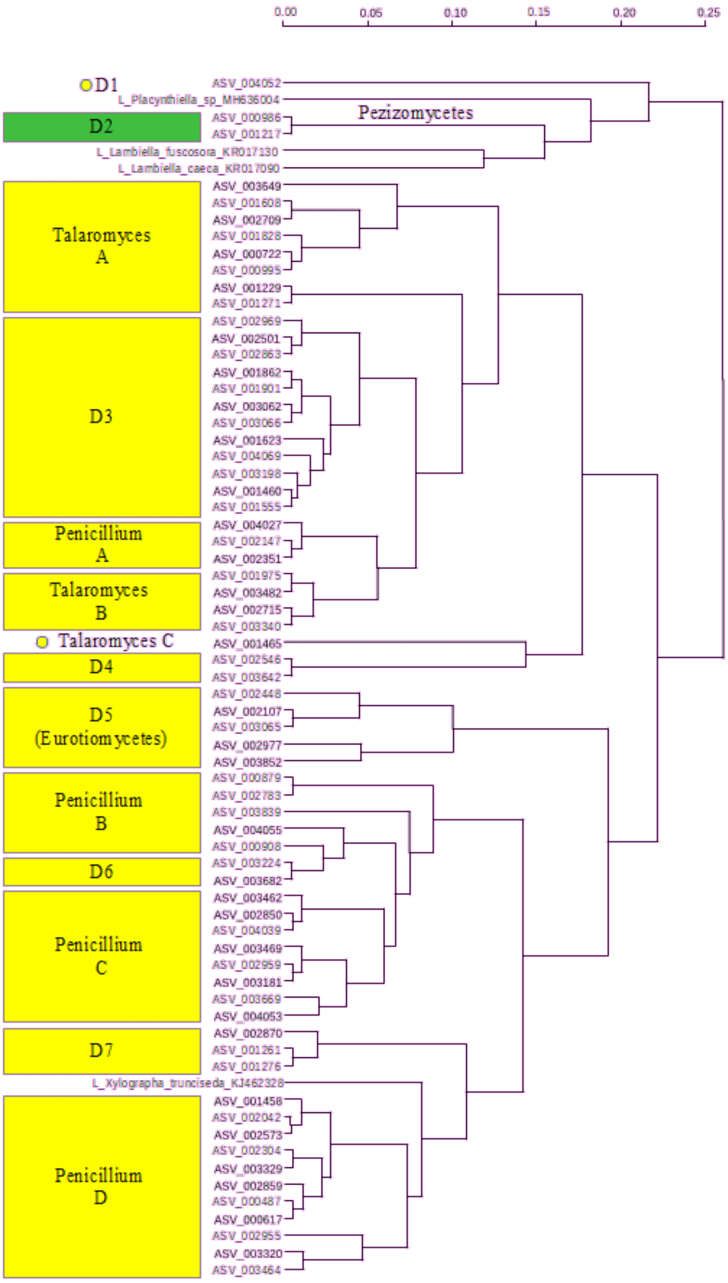

Fig. S4: Dendrogram of cluster D, consisting mainly of *Taloromyces* and *Penicillium*. The dark green cluster represents a likely lichen fungus clade, the yellow clusters can not be classified with respect to their plant pathogen potential. Dots are used for singleton sequences (see Fig. 2). Note that single species of Penicillium and Talomyces can be pathogenic to a variety of organisms. The level of difference is given in the scale.

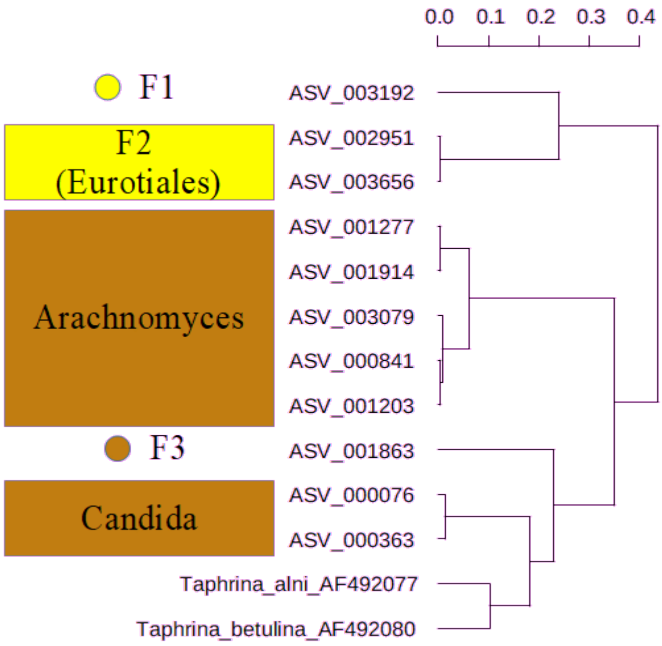

Fig. S5: Dendrogram of cluster F. The yellow clade can not be classified regarding the plant pathogen potential, the brown clades represent likely animal pathogens. Dots are used for singleton sequences (see Fig. 2). The level of difference is given in the scale.
